# Giant energy density and high efficiency achieved in bismuth ferrite-based film capacitors via domain engineering

**DOI:** 10.1038/s41467-018-04189-6

**Published:** 2018-05-08

**Authors:** Hao Pan, Jing Ma, Ji Ma, Qinghua Zhang, Xiaozhi Liu, Bo Guan, Lin Gu, Xin Zhang, Yu-Jun Zhang, Liangliang Li, Yang Shen, Yuan-Hua Lin, Ce-Wen Nan

**Affiliations:** 1 State Key Laboratory of New Ceramics and Fine Processing, School of Materials Science and Engineering, Tsinghua University, Beijing, 100084 China; 2 Beijing National Laboratory for Condensed Matter Physics, Institute of Physics, Chinese Academy of Sciences, Beijing, 100190 China; 3 Beijing National Laboratory for Molecular Science, Institute of Chemistry, Chinese Academy of Sciences, Beijing, 100190 China; 4 Collaborative Innovation Center of Quantum Matter, Beijing, 100190 China; 5 School of Physical Sciences, University of Chinese Academy of Sciences, Beijing, 100049 China

## Abstract

Developing high-performance film dielectrics for capacitive energy storage has been a great challenge for modern electrical devices. Despite good results obtained in lead titanate-based dielectrics, lead-free alternatives are strongly desirable due to environmental concerns. Here we demonstrate that giant energy densities of ~70 J cm^−3^, together with high efficiency as well as excellent cycling and thermal stability, can be achieved in lead-free bismuth ferrite-strontium titanate solid-solution films through domain engineering. It is revealed that the incorporation of strontium titanate transforms the ferroelectric micro-domains of bismuth ferrite into highly-dynamic polar nano-regions, resulting in a ferroelectric to relaxor-ferroelectric transition with concurrently improved energy density and efficiency. Additionally, the introduction of strontium titanate greatly improves the electrical insulation and breakdown strength of the films by suppressing the formation of oxygen vacancies. This work opens up a feasible and propagable route, i.e., domain engineering, to systematically develop new lead-free dielectrics for energy storage.

## Abstract (web summary)

Dielectrics with high capacitive energy storage density are essential for modern electrical devices and pulsed power systems. Here, the authors realised superior energy storage performance in lead-free bismuth ferrite-based relaxor ferroelectric films through domain engineering.

## Introduction

Dielectric capacitors are the optimal option among currently available energy storage devices to offer the highest power density (on the order of Megawatt), highest operating voltage (several hundred to thousand volts) and longest work lifetime^1–3^, which are ubiquitous and critical in modern electrical and electronic systems, especially in pulse power techniques including electrical weapon systems, hybrid electric vehicles and high-frequency inverters^4,5^. The key part in capacitors is the dielectric layer where electrostatic energy is stored in the form of electric displacement induced by an applied electric field. This unique energy storage mechanism leads to the intrinsic fast charging-discharging process and high power density. However, it also causes a relatively low energy density (~2 J cm^−3^) in comparison with fuel cells or Li-ion batteries (>20 J cm^−3^)^6,7^. Therefore, developing dielectric materials with improved energy densities is imperative to enable the reduction of size, weight, and cost of cutting-edge electrical power systems.

**Fig. 1:**
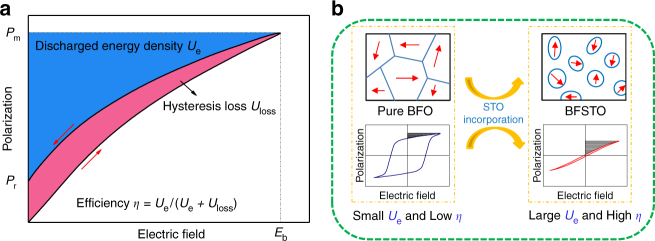
Schematic illustrations. **a** A typical *P–E* loop of a dielectric and an illustration of (discharged) energy density *U*_e_, hysteresis loss *U*_loss_ and efficiency *η*. The red arrows indicate the charging and discharging processes. **b** Schematic of domain evolution and FE-to-RFE transition induced by the incorporation of STO into BFO, leading to concurrently improved *U*_e_ and *η*. The blue outlines indicate the ferroelectric domains and the red arrows denote the spontaneous polarization directions

The energy density *U*_e_ of dielectrics, which is determined by the applied electric field *E* and the induced dielectric polarization *P*, can be mathematically expressed by

1 $$U_{\mathrm{e}} = \mathop {\int}\limits_{P_{\mathrm{r}}}^{P_{\mathrm{m}}} {E{\mathrm{d}}P}$$

where *P*_m_ and *P*_r_ are the maximum polarization and remnant polarization, respectively^4^ (Fig. 1a). Relaxor-ferroelectric (RFE) and antiferroelectric materials possess large *P*_m_ and small *P*_r_, both of which have been studied for capacitive energy storage^5^. Moreover, RFEs possess slim hysteresis loops that can be maintained at high electric fields, which results in high energy efficiency (*η*, the ratio of *U*_e_ to the total stored energy density). Currently the mainstream RFE materials for energy storage are PbTiO_3_-based ceramics because of their high dielectric permittivity and strong polarization^8^. Yet bulk ceramics bear low breakdown strengths (*E*_b_, the highest electric field a dielectric can sustain) due to the massive structural defects like pores and impurities, which hamper the realization of high energy density^9^. Recently, RFE thin films have been attracting increasing attention, where large *E*_b_ of >1 MV cm^−1^ is achievable owing to the improvement of film quality. Meanwhile, large *P*_m_ and relatively small *P*_r_ are maintained, giving rise to much elevated *U*_e_ values. For example, a *U*_e_ of ~62 J cm^−3^ at a large *E*_b_ of 3.13 MV cm^−1^ has been achieved in Bi(Ni_1/2_Ti_1/2_)O_3_-PbTiO_3_ RFE films^10^.

However, lead-containing materials pose strong threats to the environment and human health, which drives the intensive exploration of alternative lead-free materials. Some promising cases have been reported to date, among which the majority are modified BaTiO_3_ (BTO)-based RFE films. For instance, *U*_e_ values of ~37 J cm^−3^ in 0.88BaTiO_3_−0.12Bi(Mg,Ti)O_3_ films^11^ and ~52 J cm^−3^ in Ba_0.7_Ca_0.3_TiO_3_-BaZr_0__.2_Ti_0.8_O_3_ super-lattices^12^ have been reported recently. Nevertheless, there are still some drawbacks in those BTO-based RFEs that inhibit further improvements. First, the spontaneous polarization *P*_s_ of BTO is only 26 μC cm^−2^, much lower than that of PbTiO_3_ (~80 μC cm^−2^)^13^. Second, as BTO-based RFEs bears low Curie temperatures *T*_c_ (<100 °C), the deterioration of their ferroelectricity and energy storage performance can be evident at elevated temperatures^14,15^. To address these problems, we focus on a new lead-free system, namely, BiFeO_3_ (BFO), which has been acknowledged to be a promising alternative of lead-based dielectrics/ferroelectrics with a large *P*_s_ of ~100 μC cm^−2^ and a high *T*_c_ of 830 °C^16^. But pure BFO exhibits a large *P*_r_ due to strong ferroelectric (FE) hysteresis, restricting its usage for energy storage^16^. Modified BFO-based dielectrics such as BFO-BTO, BFO-(Bi_1/2_Na_1/2_)TiO_3_, and BFO-Pb(Zr,Ti)O_3_ have been reported to show RFE-like features^17–19^. Some preliminary work also found RFE properties with potentially good energy performance in BFO-SrTiO_3_ systems^20,21^. However, the underlying mechanisms for the emergence of RFE features in BFO-based dielectrics and a feasible approach to design high-energy-density BFO-based RFEs remain undiscovered.

In this contribution, we present a domain engineering method to develop BFO-based RFEs with superior energy performance. A series of solid-solution (BiFeO_3_)_1-*x*_–(SrTiO_3_)_*x*_ (denoted as BFSTO, 0 < *x* < 1) films are designed and fabricated. It is revealed that the incorporation of SrTiO_3_ (STO) can transform the micrometer-scale FE domains of BFO into highly-dynamic polar nano-regions (PNRs), leading to a macroscopic FE-to-RFE transition (schematically shown in Fig. 1b). The favourable RFE state induced by domain engineering possesses simultaneously large *P*_m_ and suppressed *P*_r_, which, together with the enhanced breakdown strength with the STO incorporation, gives rise to giant energy densities with high energy efficiency of the BFSTO films.

## Results

### Microstructure

**Fig. 2:**
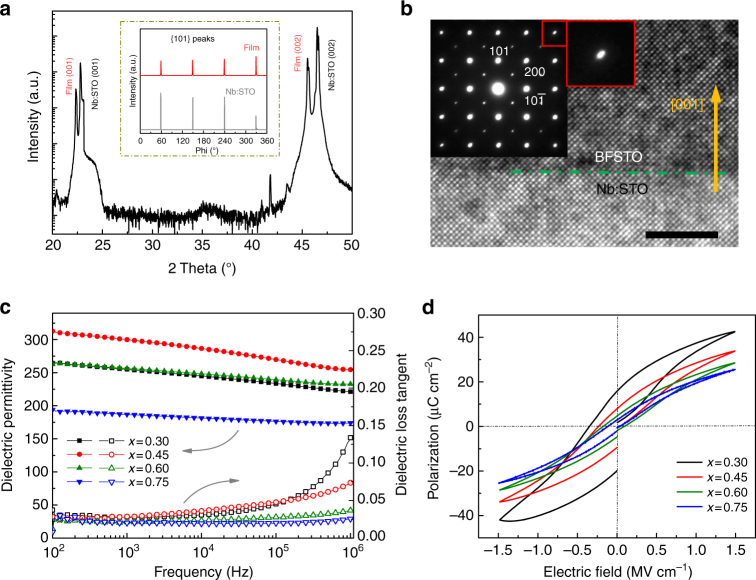
Microstructure, dielectric, and ferroelectric properties. **a** XRD pattern of the BFSTO film with *x* = 0.45; the insets shows the phi scans of its {101} planes corresponding to the Nb:STO substrate. **b** A high-resolution TEM image of the interface of BFSTO film with *x* = 0.45 and Nb:STO substrate (scale bar: 5 nm); the inset is the SAED pattern at the interface zone. **c** Frequency-dependent dielectric permittivity and loss tangent and **d** bipolar *P*–*E* loops of the BFSTO films

The BFSTO films were grown on the 0.7 wt% Nb-doped STO (Nb:STO) single crystal substrates using a pulsed laser deposition (PLD) system. X-ray diffraction (XRD) patterns prove the highly preferential *c*-axis orientation of the films as revealed by the (00*l*) peaks without any trace of others (Fig. 2a and Supplementary Fig. 1). Phi scan of the {101} planes (inset of Fig. 2a and Supplementary Fig. 1) further reveals that the films inherit a four-fold rotational symmetry from the substrate: (001)_BFSTO_ // (001)_Nb:STO_ (out of plane) and [010]_BFSTO_ // [010]_Nb:STO_ (in plane), indicating an excellent cubic-on-cubic epitaxy nature. The BFSTO films have a smooth surface with an average roughness of ~1 nm, as shown by an atomic force microscope image (Supplementary Fig. 2). A low-magnification high-angle angular dark-field (HAADF) image (Supplementary Fig. 3) displays clearly the sandwich structure consisting of the Nb:STO substrate, BFSTO film and Au top electrode. The BFSTO films are dense and crack-free, indicating good film quality due to the epitaxial growth. A direct evidence of the epitaxy is provided by a high-resolution transmission electron microscope (TEM) image (Fig. 2b) at the Nb:STO/BFSTO (*x* = 0.45) interface, which shows that both the film and matrix have single-crystal nature with a heteroepitaxial interface. The selected area electron diffraction (SAED) at the interface (inset of Fig. 2b) reveals that the BFSTO film shares the same type of diffraction pattern with the substrate. A small distortion of the diffraction spots shown in the red frame can be noticed because of the slight difference of lattice parameters between the BFSTO film and the Nb:STO substrate.

### Dielectric and FE properties

Dielectric permittivity and loss tangent of the BFSTO thin films depending on frequency are displayed in Fig. 2c. It is interesting to note that the dielectric permittivity reaches the maximum of ~300 (at 1 kHz) in the film with *x* = 0.45, which can be attributed to the rhombohedral-to-pseudo cubic phase boundary, in accordance with the findings in BFSTO bulk ceramics^22^. The permittivity declines gradually due to further increase of the paraelectric STO content, along with the loss tangent substantially reduced to 0.037 and 0.025 (at 1 MHz) for *x* *=* 0.60 and 0.75, respectively, which are at the same level with the best results reported in FE films, e.g. ~0.05 for PLZT film^23^ and ~0.03 for Ba(Zr,Ti)O3 film^24^. The low loss tangent can prevent the self-heating and thermal runaway of dielectrics, which is crucial to the BFSTO films for capacitor applications^25,26^. Another remarkable feature of the BFSTO films is the tailoring of FE properties with the incorporation of STO. Bipolar *P*–*E* hysteresis loops of the films measured with an electric field of 1.5 MV cm^−1^ are shown in Fig. 2d. One can see that the film with *x* = 0.30 possesses the largest *P*_m_ and *P*_r_ with remarkable hysteresis, indicating typical FE properties. Notably, films with increasing *x* exhibit much narrowed *P–E* loops with substantial reduction of *P*_r_, which represents a crossover towards RFE feature. The temperature-dependent dielectric spectroscopies of the BFSTO films exhibit broadened dielectric peaks and frequency dispersion (Supplementary Fig. 4), further evidencing the RFE characteristics^14^.

### Energy storage performance

**Fig. 3:**
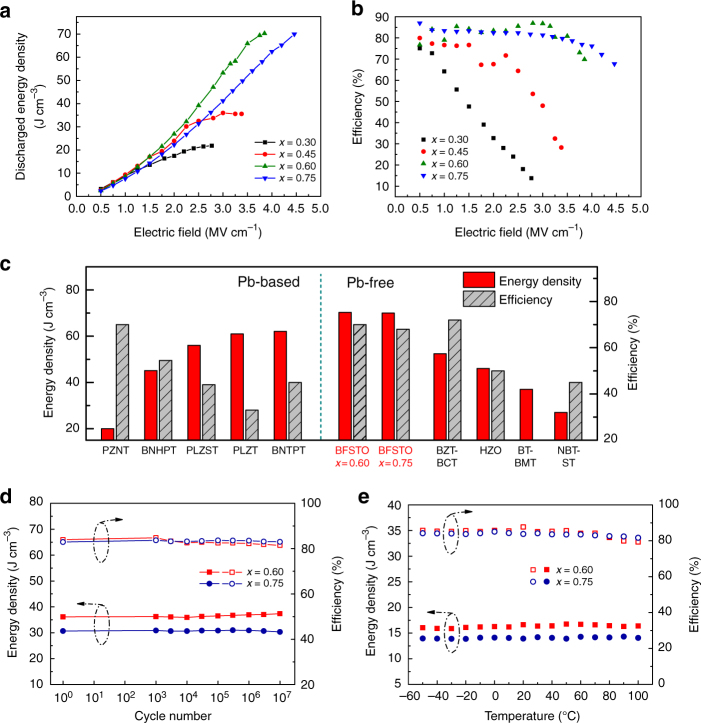
Energy storage performance. **a** Discharged energy density and **b** efficiency of the BFSTO films as a function of the applied electric field. **c** Comparisons of energy density and efficiency between BFSTO and representative dielectric systems^10–12,28–33^, showing that the BFSTO films possess the most outstanding energy storage performance. **d** Energy density and efficiency for *x* = 0.60 and 0.75 at an electric field of 2.5 MV cm^−1^ over 1 × 10^7^ charging-discharging cycles. **e** Temperature-dependent energy storage performance for *x* = 0.60 and 0.75 at an electric field of 1.5 MV cm^−1^

The discharged energy densities of the BFSTO films are determined from their unipolar *P-E* loops (Supplementary Fig. 5) and plotted in Fig. 3a. It is most striking that the film with *x* = 0.60 exhibits a giant *U*_e_ of 70.3 J cm^−3^ at 3.85 MV cm^−^^1^; the film with *x* = 0.75 also achieves a *U*_e_ of 70.0 J cm^−3^ at 4.46 MV cm^−1^. Although the films with *x* = 0.45 or 0.30 possess higher *P*_m_ values, they also bear larger *P*_r_ (Supplementary Fig. 5), probably because of the larger FE hysteresis and higher leakage current at high electric fields^27^. As a result, their energy densities are substantially limited. For example, the film with *x* = 0.30 shows a maximum *U*_e_ of only 21.8 J cm^−3^ at 2.75 MV cm^−1^, which is even smaller than that (36.1 J cm^−3^) of *x* = 0.75 at the same electric field. The large *P*_r_ also causes remarkable decline of energy efficiency *η* of *x* = 0.30 and 0.45, as shown in Fig. 3b. In sharp contrast, high *η* values of 70% at 3.85 MV cm^−1^ and 68% at 4.46 MV cm^−1^ are maintained for *x* = 0.60 and 0.75, respectively. Note that high efficiency is also crucial for dielectrics because it means less waste heat, better reliability and longer lifetime of capacitors in practical applications. The energy densities and efficiency of the BFSTO films are compared with those of representative lead-based and lead-free material systems reported previously, as displayed in Fig. 3c^10–12,28–33^. The results evidently show that the energy densities of the BFSTO films are superior to those of other reported lead-free systems (35% over the best BTO-based systems^12^) and rival the lead-based materials. Additionally, the concomitantly achieved high efficiency makes the BFSTO films more attractive for energy storage applications.

Long-term working stability is another significant requirement for dielectrics. Fast charging-discharging cycling test has been conducted with a triangle electric field of 10 kHz, 2.5 MV cm^−1^. It can be clearly seen in Fig. 3d that the films with *x* = 0.60 and 0.75 exhibit little degradation of either *U*_e_ or *η* over 1 × 10^7^ cycles. The excellent stability can be ascribed to (1) the robust mechanical strength of the films that prevents mechanical damage from electrostatic force and (2) the nearly defect-free microstructure and highly-dynamic RFE feature that inhibit domain wall pinning during the repeated polarization switching^34^. Thermal stability of the energy performance of BFSTO films was also investigated. The results (Fig. 3e) demonstrate that both the films with *x* = 0.60 and 0.75 possess highly stabilized energy density and efficiency in a wide temperature range of –50 to 100 °C, which meets well with the requirement for applications in harsh environment such as aerospace and hybrid electric vehicles. The films with *x* = 0.30 and 0.45, on the contrary, show deterioration of energy performance at slightly elevated temperatures (Supplementary Fig. 6). This should be pinned on the increase of electrical conduction loss and similar phenomena have been reported in polymer and ceramic dielectrics^6,35^. The conduction loss and FE loss of the BFSTO films at various temperatures are decoupled based on the method by Khanchaitit et al.^36^, as shown in Supplementary Fig. 7 and Supplementary Note 1. It is found that the suppression of conduction loss for *x* = 0.60 and 0.75 is the main contributor to the thermal stabilization of energy performance.

### FE-to-RFE transformation and domain evolution

**Fig. 4:**
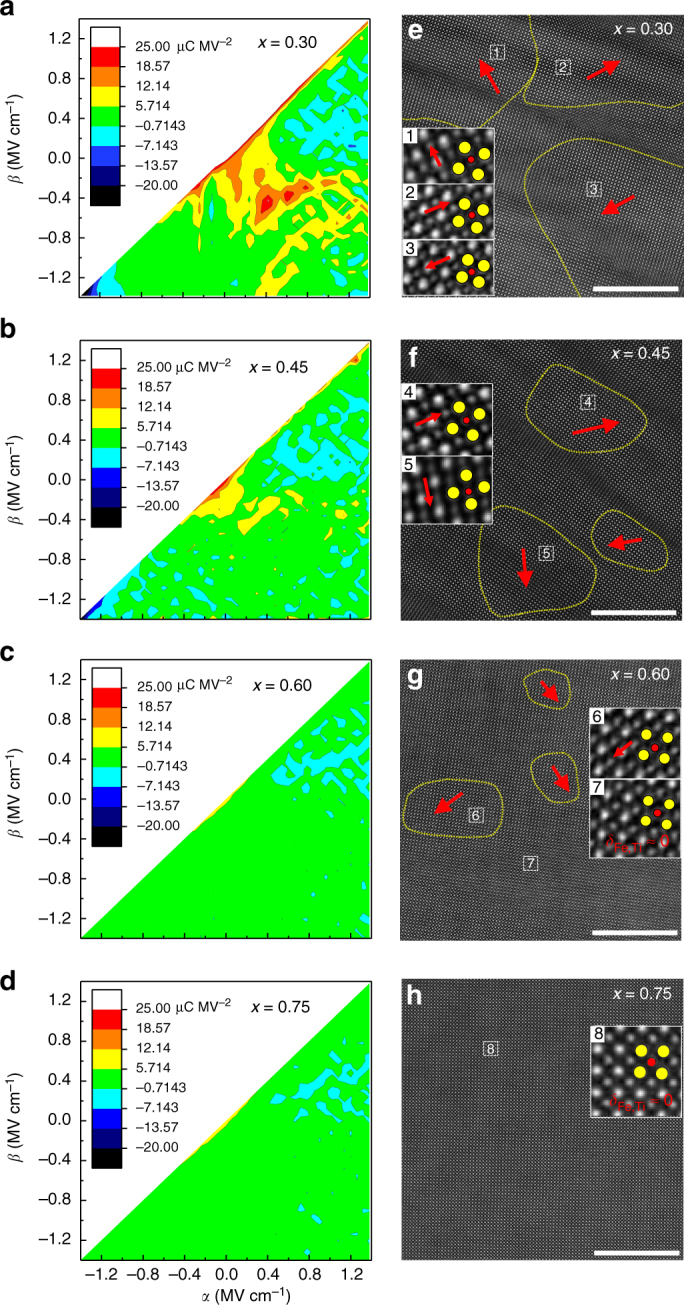
Evolution of FORC distribution and domain configuration. **a**–**d** FORC distribution *p*(*α*,*β*) and **e**–**h** HAADF-STEM images of the atomic-scale ferroelectric domain structure of the BFSTO films (scale bars: 10 nm). The yellow dashed lines in **e**–**h** mark the domains with spontaneous polarization and the red arrows denote the Fe/Ti ion displacement (*δ*_Fe/Ti_) directions. The insets of **e**–**h** are magnified images of selected areas to show the *δ*_Fe/Ti_, where the yellow and red circles denote the Bi/Sr and Fe/Ti ion columns, respectively

The outstanding energy performance of the BFSTO films should be ascribed to their favourable RFE properties. On the one hand, due to the existence of highly-polarizable BFO component, large *P*_m_ values are maintained, e.g., ~59.2 μC cm^−2^ for *x* = 0.60, representing at least 50% improvement over those of BTO-based RFEs (<40 μC cm^−2^)^11,12^ and thus guarantees an increased *U*_e_. On the other hand, the FE-to-RFE transformation markedly suppresses the *P*_r_ values, e.g., ~11.1 μC cm^−2^ for *x* = 0.60, hence inducing the minimized hysteresis loss and high *η*. In order to gain more insights into the FE-to-RFE transformation, first order reversal curve (FORC) distributions of the BFSTO films are investigated, which is a sensitive method to give reasonable descriptions of hysteresis behaviors^37^. The FORC method is based on the Preisach model^38,39^ assuming that a hysteresis loop consists of a set of rectangular loops (termed as “hysterons”) characterized by the positive-switching coercive field *α* and the negative-switching coercive field *β* (*β* ≤ *α*, schematically shown in Supplementary Fig. 8). The hysterons have a distribution in regard to *α* and *β*, which is the FORC distribution *p*(*α, β*)^39,40^. In this work, the *p*(*α, β*) diagrams of the films were derived from a series of FORC loop measurements (Supplementary Figs. 8 and 9). Based on the evolution of the diagrams shown in Figs 4a–d, the crossover from FE towards RFE state with increasing STO content has been illustrated. The diagram of the film with *x* = 0.30 (Fig. 4a) exhibits a high-intensity distribution zone located near the origin point, which represents the switching of FE domains at lower electric fields, and is responsible for the large total polarization of the film^41^. The *p*(*α*, *β*) intensity at high-electric-field zones, however, is substantially suppressed due to the saturation of domain switching. This inhomogeneous distribution reflects the typical FE characteristics with strong polarization nonlinearity^38^. As *x* increases to 0.45 (Fig. 4b), the high-intensity zones significantly shrink toward the origin point, indicating smaller coercive fields and decreased nonlinearity. This suggests a shift toward RFE state, since RFE is characterized with small energy barrier for switching and ultralow coercivity. When *x* further increases to 0.60 and 0.75 (Fig. 4c,d), *p*(*α*, *β*) becomes more evenly distributed in the whole electric field range, indicating further weakened FE nonlinearity and dispersed polarization contribution in the RFE state^40^.

From a microscopic perspective, the RFE properties are proposed to be linked with the emergence of PNRs, which have been observed in both lead-based and lead-free RFE materials^8,42^. Theoretical simulations have offered clear insights into the existence of PNRs in the Ba(Zr,Ti)O_3_ system^43^, and the evolution of PNRs under electric fields that causes RFE properties^44^. Accordingly, it is proposed that the incorporated STO into BFO (Sr into Bi sites and Ti into Fe sites) induces compositional and chemical disorder in the BFSTO films, breaking the long-range FE order of BFO and transforming the FE domains into PNRs, thus resulting in a RFE state^45^. The FE domains of pure BFO films can be observed with the piezoelectric force microscope (PFM) technique (Supplementary Fig. 10), which are of several hundred nanometer to several micrometer and strongly coupled with each other. To validate the emergence of PNRs in the RFE BFSTO films, atomic-scale characterizations based on the scanning transmission electron microscopy (STEM) is employed^46^. In the HAADF *Z*-contrast STEM images shown in Figs 4e–h, the A-site ion columns (Bi^3+^ and Sr^2+^) appear as the brighter dots while B-site ion columns (Fe^3+^ and Ti^4+^) show weaker contrast. The displacements of Fe/Ti ions relative to the lattice center can be directly observed in the images (see the magnified zones in the insets). The spontaneously polarized zones, i.e., the PNRs are schematically marked with yellow dashed lines, and the red arrows show the directions of Fe/Ti ion displacement vector *δ*_Fe/Ti_. One can first note the decrease tendency of the area of long-range polarization order with increased STO content, which means a weakened ferroelectricity and is in accordance with Fig. 2d. As for the domain configuration, the results demonstrate an apparent picture that large-sized domains transform gradually into nano-scale PNRs as the STO content increases. The distance between neighboring PNRs also increases, implying weakened inter-coupling and enhanced domain switching mobility^40,47^. It should be pointed out that the domain structure of the film with *x* = 0.75 is hard to distinguish, probably because the PNR sizes become so small that the ion displacements are submerged by the average effect of ion column projection. Besides the STEM images, the domain evolution in the BFSTO films can be also verified by the characterization of domain switching with PFM. As shown in Supplementary Fig. 11, pure BFO film exhibits stable retention of polarization at least 10 h after being poled with a voltage of 20 V. In stark contrast, in the film with *x* = 0.60, the polarized domains switch back mostly in 30 min after the applied voltage is removed. This phenomenon verifies the much enhanced domain mobility in the BFSTO films, indicating the occurrence of domain evolution^48^.

### Breakdown strength

**Fig. 5:**
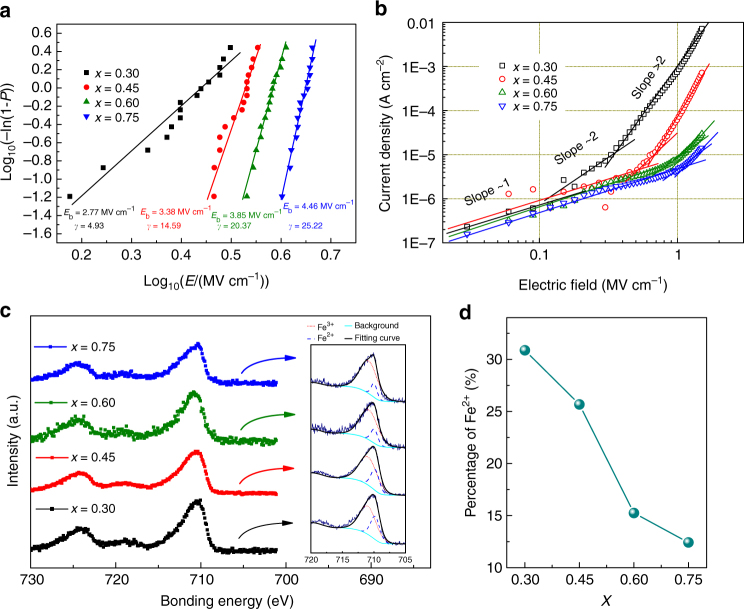
Breakdown strength and electric conduction. **a** Two-parameter Weibull Distribution analysis of dielectric breakdown strengths, **b** room-temperature leakage current densities as a function of biased electric field, **c** XPS fitting for Fe valences and **d** percentage of Fe^2+^ out of all Fe ions in the BFSTO films

Improved breakdown strength is another important factor for the superior energy performance of the BFSTO films since it allows a larger applied electric field and full polarization. The characteristic breakdown field *E*_b_ of the films was obtained using a two-parameter Weibull distribution fitting^7^, as plotted in Fig. 5a. Notably, the BFSTO films exhibit much improved breakdown strengths that are positively related to the STO content. The *E*_b_ values of the films with *x* = 0.60 and 0.75 reach 3.85 MV cm^−^^1^ and 4.46 MVcm^−1^, respectively, which represent a nearly twofold enhancement over that of pure BFO film (~1.5 MV cm^−1^,)^49^ and are among the highest values in ceramic films^12,32,33^. Moreover, the Weibull modulus *γ* that evaluates the scatter of breakdown field data also increases with the STO content. For example, *γ* = 4.93 and 25.22 for *x* = 0.30 and 0.75, respectively, suggesting a narrowed distribution of *E*_b_ values, namely, an improvement of dielectric reliability^36^. The pronounced enhancement of breakdown performance is reasonably ascribed to the improved electric insulation in the BFSTO films^5^. Figure 5b shows that the steady-state leakage current *J* of the BFSTO films is reduced by several orders of magnitude with increased STO content, e.g., from 7.2 × 10^–3^ A cm^–2^ for *x* = 0.30 to 1.4 × 10^−5^ A cm^−2^ for *x* = 0.75 at a bias field of 1.5 MV cm^−1^. With a closer look at the *J*–*E* curves, one can notice that all samples follow Ohm’s law at low electric fields (log_10_*J* ~ *S* log_10_*E*, where the slope *S* ≈ 1). As the electric field exceeds a critical value *E*_0_, new conduction mechanisms with various sources of charge carriers become dominant, which in our case are the space charge limited conduction (log_10_*J* ~ *S* log_10_*E*, where *S* ≈ 2) at medium fields and the Schottky/Poole-Frenkel emission at high fields^50,51^. The specific *E*_0_ values are 0.13, 0.52, 0.81, and 1.05 MV cm^−1^ for *x* = 0.30, 0.45, 0.60, and 0.75, respectively, which indicates that the ability of inhibiting new charge carriers is enhanced by incorporating STO. As a matter of fact, the main charge carriers in BFO-based materials are acknowledged to be oxygen vacancies induced by the chemical valence fluctuation of Fe ions^49^. The corresponding reaction is written as

2 $$2{\mathrm F{\mathrm e}}_{\mathrm F{\mathrm e}} + {\mathrm O}_{\mathrm O} \to 2{\mathrm F}{\mathrm e}_{{\mathrm F}{\mathrm e}}\prime + {\mathrm V}_{\mathrm O}^{\cdot \cdot } + \frac{1}{2}{\mathrm O}_2$$

Hence a straightforward way to reduce the leakage current is to inhibit the Fe^3+^-to-Fe^2+^ transition, which has been proved effective in BFO-based ceramics with partially substitution of analogous ions (such as Ti, Mn, Co, and Al etc.) at Fe sites^49,52^ or BFO-ABO_3_ (such as PbTiO_3_ and BTO) solid solutions^53,54^. In the BFSTO system, we reveal that the Fe^3+^-to-Fe^2+^ transition is also suppressed by the incorporation of STO. The valence states of Fe ions are determined by X-ray photoelectron spectroscopy (XPS), as shown in Fig. 5c. The Fe 2*p*_3/2_ peaks are fitted by Lorentzian-Gaussian functions and the peaks corresponding to Fe^2+^ and Fe^3+^ are located at 709.9 eV and 711.1 eV, respectively^52^. The percentage of Fe^2+^ is calculated according to the integration of the fitted peaks. The results (Fig. 5d) clearly demonstrate the decline of Fe^2+^ ratio from 31 to 12% as the STO content increases from 0.30 to 0.75, indicating the significant stabilization of Fe valence and suppressed oxygen vacancies. The electric insulation of the BFSTO films is therefore improved, which further leads to the enhancements of breakdown strength and energy performance for the BFSTO films.

## Discussion

We present a feasible domain engineering method that enables the development of new lead-free RFE materials and the realization of high-performance capacitive energy storage. It is revealed by HAADF-STEM that the long range FE order in BFO is interrupted by the incorporation of STO, resulting in the transformation of micron-scale FE domains into nano-scale PNRs. At the macroscopic level, the FE state with large hysteresis loss transits into a RFE state that maintains relatively high polarization but exhibits much suppressed hysteresis and remnant polarization. The favorable RFE property, together with the enhanced breakdown strengths, gives rise to giant energy storage densities of ~70 J cm^−3^ in the BFSTO films with both *x* = 0.60 and 0.75, which are superior to other reported lead-free systems and rival the best lead-based systems. This corresponds with the first-principle simulations by Xu et al.^55,^ proving the huge potential of BFO-based materials as high-energy-density dielectrics. It may be noted that the simulations predicted even higher energy density (100–150 J cm^−3^), indicating that there is still much room for further improvement in the BFSTO system.

The domain engineering technique proposed in our work is not only efficient in achieving targeted properties but also propagable in applications. Note that some other existing domain engineering methods, such as inducing anisotropy and stress in FE films by selected substrate^56,57^, or poling FE single crystals with selective directions to form a multi-domain configuration^8^, are actually limited by material categories and geometry parameters. As for the technique in this work, it can be readily applied in ceramics, single crystals as well as films. Moreover, it can be extended to the development of a number of other possible BFO-based RFEs such as BFO-CaTiO_3_, BFO-BaZrO_3_, and BFO-BaSnO_3_ etc. We can also expect to obtain series of new RFE systems with domain engineering by incorporating the above analogous ABO_3_ components into other FEs such as (K,Na)NbO_3_, LiNbO_3_, and LiTaO_3_ etc. Considering that the mentioned systems have been seldom fabricated or investigated, we hope that the paradigm demonstrated in this contribution could motivate more efforts to shed light on new lead-free RFE materials and to develop promising candidates for dielectric energy storage.

## Methods

### Film fabrication

The BFSTO ceramic targets were sintered by a conventional solid state reaction method. To compensate the Bi evaporation during target sintering and film deposition, 10 mol% excess Bi was added. The BFSTO films were fabricated using a PLD technique. A KrF laser excimer (λ = 248 nm) with a repetition rate of 10 Hz was employed and the laser energy density was ~1.7 J cm^−2^. The optimal deposition parameters were set to be 700 °C with an oxygen pressure of 1.3 Pa. After deposition, the films were annealed at 500 °C with an oxygen pressure of 500 mbar for 30 min, and then cooled down to room temperature at a speed of 10 °C min^–1^. The thicknesses of the films are ~500 nm. Circular Au top electrodes (300 μm in diameter and ~100 nm in thickness) were sputtered through a shadow mask to form the capacitor structures.

### Characterization

The crystallinity and epitaxy properties of the BFSTO films were characterized using an X-ray diffractometer (XRD; Rigaku-2500). Surface and FE domain morphologies were revealed by an atomic force microscope (AFM, MFP 3D infinity, Oxford Instruments); and the cross-sectional image of the films were captured by a TEM, Philips CM200. The atomic interface morphology and domain structure images of the BFSTO films were obtain using a high-resolution scanning transmission electron microscope (STEM, JEOL ARM200CF). XPS measurements were conducted on a Thermo XPS ESCALAB 250Xi instrument (Thermo Scientific). Room-temperature dielectric permittivity and loss were measured with a precision impedance analyzer (HP 4294A, Agilent) with a perturbation voltage of 500 mV. Measurements of FE hysteresis loops, DC leakage current and FORC loops were performed on a ferroelectric test system (Precision Multiferroic II, Radiant Technologies, Inc.).

### The FORC distribution

The distribution is derived from a series of FORC loops measurement. During the measurement, the FE film is exposed to a half-sinusoidal electric field that increases from the negative saturation field –*E*_max_ to *α*_*i*_ (–*E*_max_ + *i*Δ*E*) and then decreases to *β*_*j*_ (−*E*_*max*_ + *j*Δ*E;*  *j* ≤ *i*, Supplementary Fig. 8), the measured polarization *P*(*α*_*i*_, *β*_*j*_) obeys the following equation^32,33^:

3 $$P\left( {\alpha _i,\beta _j} \right) - P\left( {\alpha _i,\alpha _i} \right) = - 2\mathop {\int } \limits_{\beta _j}^{\alpha _i} \left(\mathop {\int }\limits_{\beta _j}^{\alpha _i} p\left( {\alpha ,\beta } \right){\mathrm d}\alpha \right){\mathrm d}\beta - 2\mathop {\int }\limits_{\beta _j}^{\alpha _i} p_{\mathrm{rev}}(\alpha ){\mathrm d}\alpha$$

where *p*(*α*,*β*) is the irreversible distribution and *p*_rev_(*α*) is the reversible distribution. Here, only the irreversible distribution is investigated because it gives the main information of FE hysteresis and nonlinearity. Differentiation of Eq. (3) with respect to *α*_*i*_ and *β*_*j*_ gives

4 $$\frac{{\partial ^2P\left( {\alpha _i,\beta _j} \right)}}{{\partial \alpha _i\partial \beta _j}} = 2p(\alpha _i,\beta _j)$$

In this work, *E*_max_ is set to be 1.5 MV cm^−1^, Δ*α* = Δ*β* = Δ*E* = 0.06 MV cm^−1^. An approximate method to calculate *p*(*α*, *β*) is

5 $$p\left( {\alpha ,\beta } \right) \approx \frac{1}{2}\frac{{P\left( {\alpha ,\beta + \Delta \beta } \right) - P\left( {\alpha ,\beta } \right) - P\left( {\alpha - \Delta \alpha ,\beta + \Delta \beta } \right) + P\left( {\alpha - \Delta \alpha ,\beta } \right)}}{{\Delta \alpha \Delta \beta }}$$

In this way we get a discrete distribution *p*(*α*, *β*), which in fact represents the distribution of hysterons in the zone of *α*_*i*_ – Δ*α* ≤ *α* ≤ *α*_*i*_ and *β*_*j*_ − Δ*β* ≤ *β* ≤ *β*_*j*_ + Δ*β* for *p*(*α*, *β*) = *p*(*α*_*i*_, *β*_*j*_) (*j* ≤ *i*)^32^.

### Weibull distribution

A two-parameter Weibull distribution function of the electric breakdown strength is presented as

6 $$P(E) = 1 - {\mathrm{exp}}\left( { - (E/E_{\mathrm b})^ \wedge \gamma } \right)$$

where *P*(*E*) is the cumulative probability of electric breakdown, *E* is the measured breakdown field, *E*_b_ is the characteristic breakdown field corresponding to a 63.2% probability of failure and *γ* is the Weibull modulus. For sample 1, 2…*n*, *E*_*i*_ is the breakdown field of the *i*th sample which is ranked in an increasing order. *P*(*E*)_*i*_ = *i*/(*n* + 1) is numerically regarded as the cumulative probability of electric failure. Therefore, Eq. (6) transforms into log_10_(–ln(1 – *P(E)*_*i*_)) ~ *γ* log_10_(*E*_*i*_), where *γ* is the slope. In this work, 15 measurements of each composition were conducted for the Weibull fitting, i.e., *n* = 15.

### Data availability

All relevant data are available from the corresponding author upon reasonable request.

## Electronic supplementary material

Supplementary Information
