# Supplementary material for: Giant energy density and high efficiency achieved in bismuth ferrite-based film capacitors via domain engineering
Source: Nat Commun. 2018 May 8;9:1813. doi: 10.1038/s41467-018-04189-6 (PMC5940880; doi:10.1038/s41467-018-04189-6)
Supplement: Supplementary file 1 — Supplementary Information [file 41467_2018_4189_MOESM1_ESM.pdf]

Supplementary Information

Giant energy density and high efficiency achieved in Bismuth Ferrite-based film capacitors via domain engineering

Pan et al.

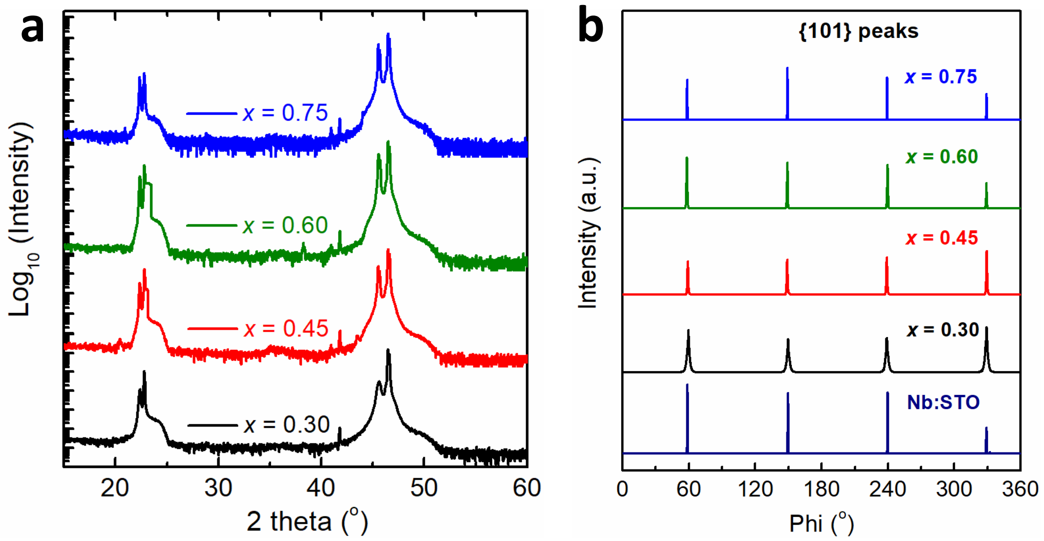

Supplementary Figure 1. X-ray diffraction patterns. (**a**) Theta - 2 theta scan pattern of the BFSTO (x = 0.30, 0.45, 0.60 and 0.75) solid-solution films epitaxially grown on the (001) Nb:STO single crystal substrates. (**b**) Phi scans of {101} planes of the BFSTO films and the Nb:STO substrate.

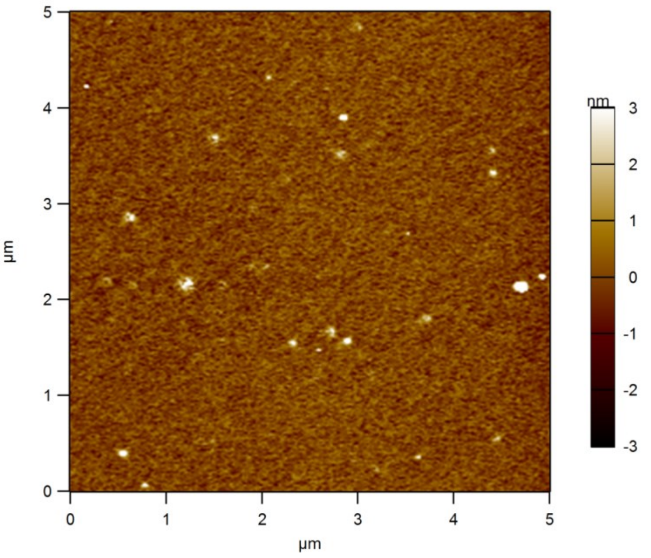

Supplementary Figure 2. Atomic force microscope (AFM) image. An AFM image of the surface morphology of the BFSTO film with x = 0.45, indicating the small roughness of ~ 1 nm.

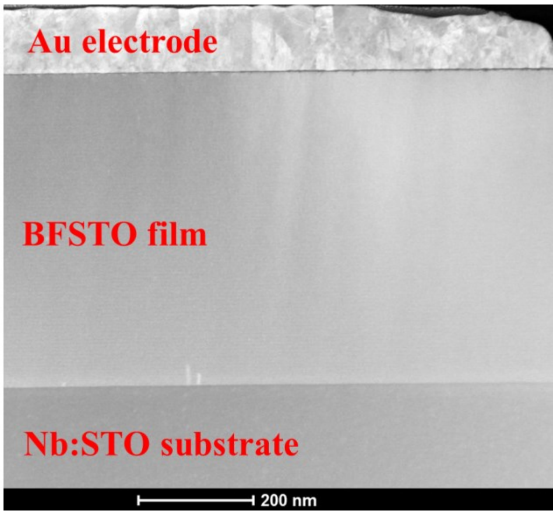

Supplementary Figure 3. Low-magnification high-angle angular dark-field (HAADF) image. A HAADF image of the cross-sectional sandwich structure of the BFSTO film capacitor with x = 0.45, indicating the dense and crack-free microstructure.

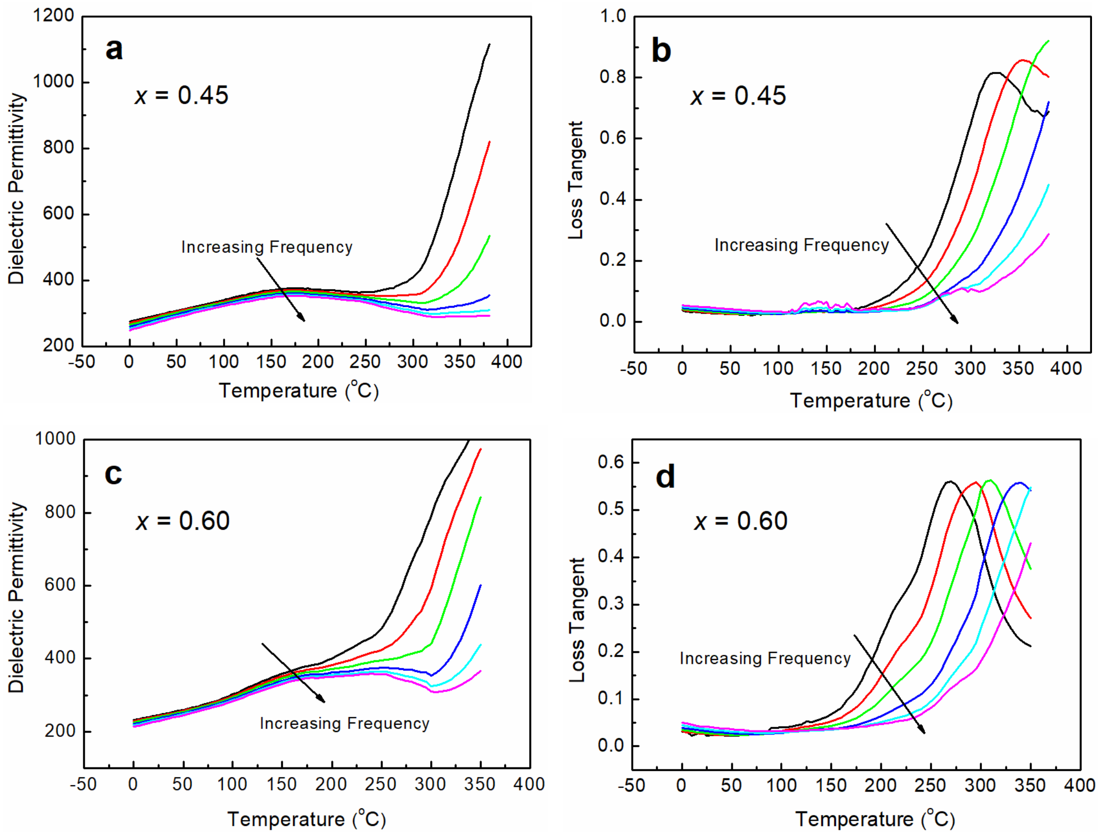

Supplementary Figure 4. Temperature-dependent dielectric properties. The temperature dependence of dielectric permittivity of (**a**) $x = 0.45$, (**c**) $x = 0.60$ and dielectric loss tangent of (**b**) $x = 0.45$, (**d**) $x = 0.60$. The measurement frequencies are 5, 10, 20, 50, 100 and 200 kHz, respectively. The broadened permittivity peaks correlated to the ferroelectric-paraelectric phase transition and the frequency dispersion of the permittivity and loss tangent indicate typical relaxor characteristics of the BFSTO films.

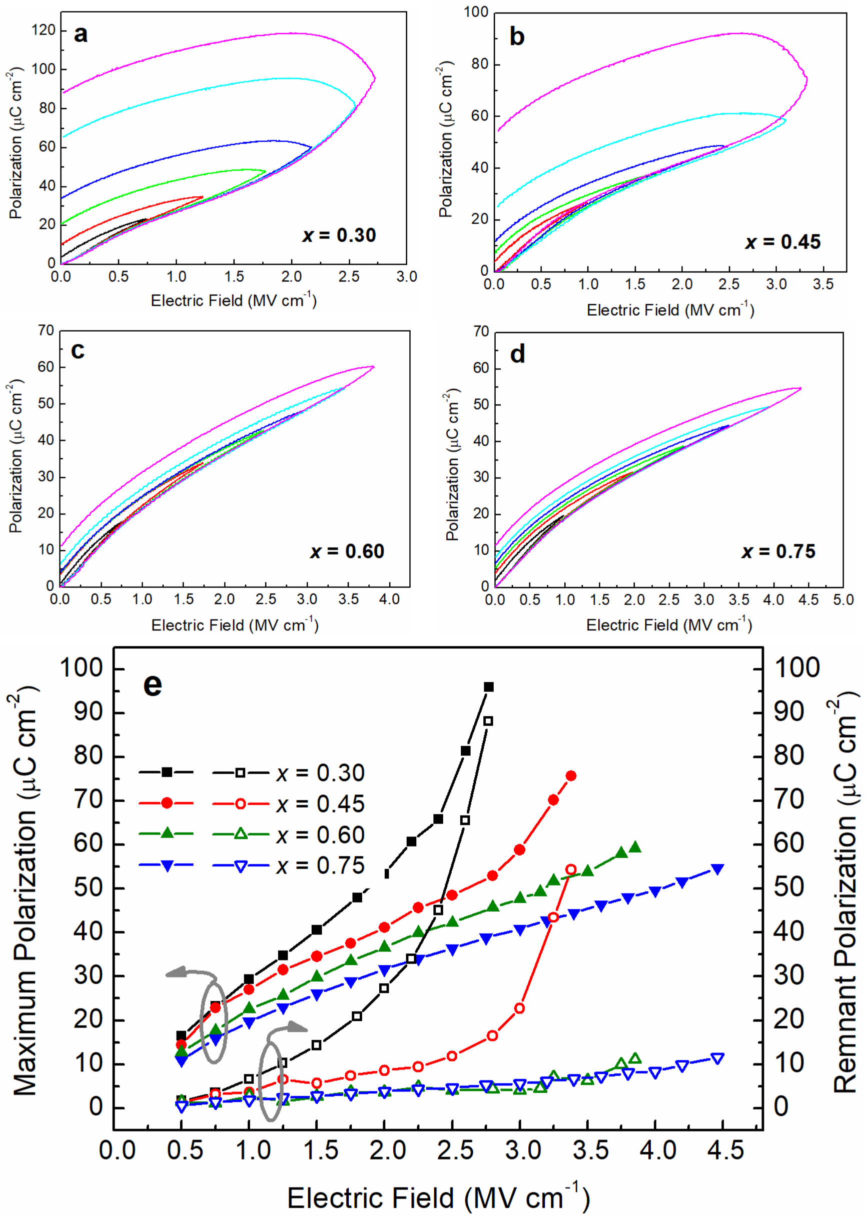

Supplementary Figure 5. Polarization – electric field (*P-E*) hysteresis loops. Unipolar *P-E* loops of BFSTO films at various electric fields up to their breakdown fields. (**a**) x = 0.30, (**b**) x = 0.45, (**c**) x = 0.60 and (**d**) x = 0.75; (**e**) The maximum polarization and remnant polarization of the BFSTO films as a function of the electric field.

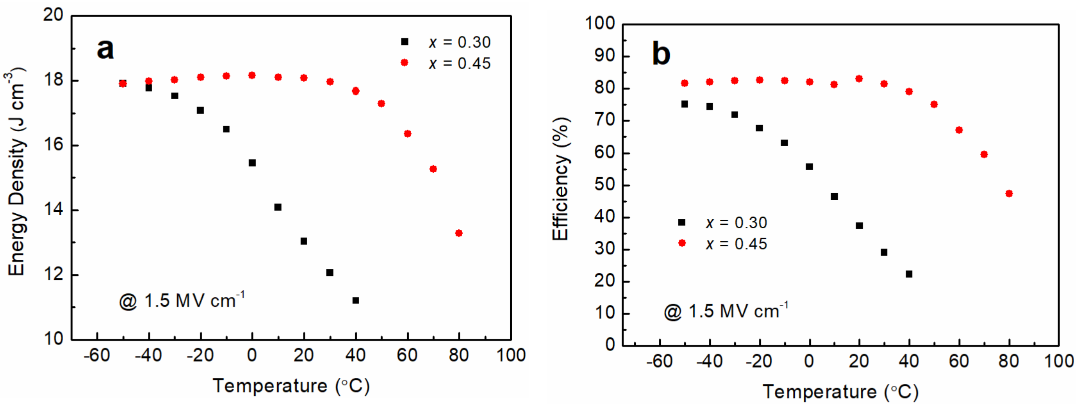

Supplementary Figure 6. Temperature dependence of energy storage performance. (**a**) Energy density and (**b**) energy efficiency of the BFSTO films with $x = 0.30$ and 0.45 at various temperatures.

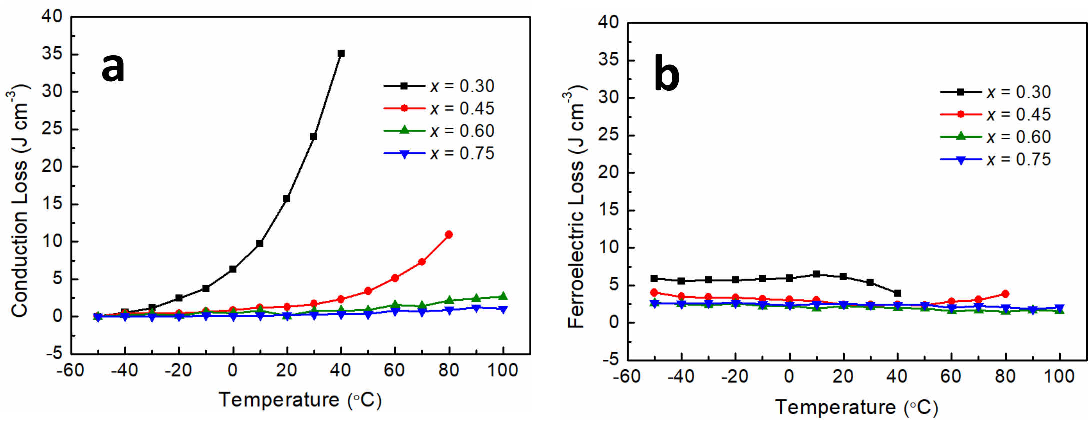

Supplementary Figure 7. Temperature-dependent conduction and ferroelectric loss. (**a**) Conduction loss and (**b**) ferroelectric loss of the BFSTO films at various temperatures with an electric field of 1.5 MV cm^{-1}.

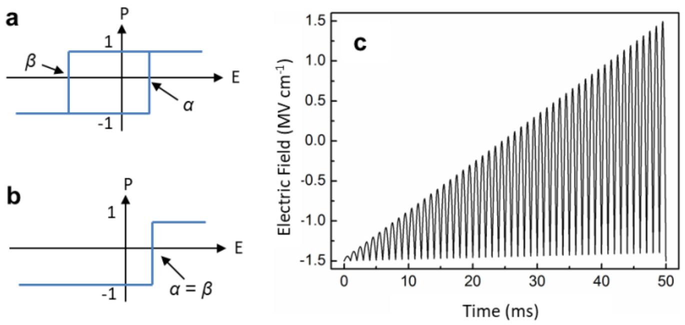

Supplementary Figure 8. Schematic illustration of hysteron and FORC measurement. (**a**) Irreversible hysteron, (**b**) reversible hysteron and (**c**) the set of biased half-sinusoidal electric field used for the measurement of FORC loops.

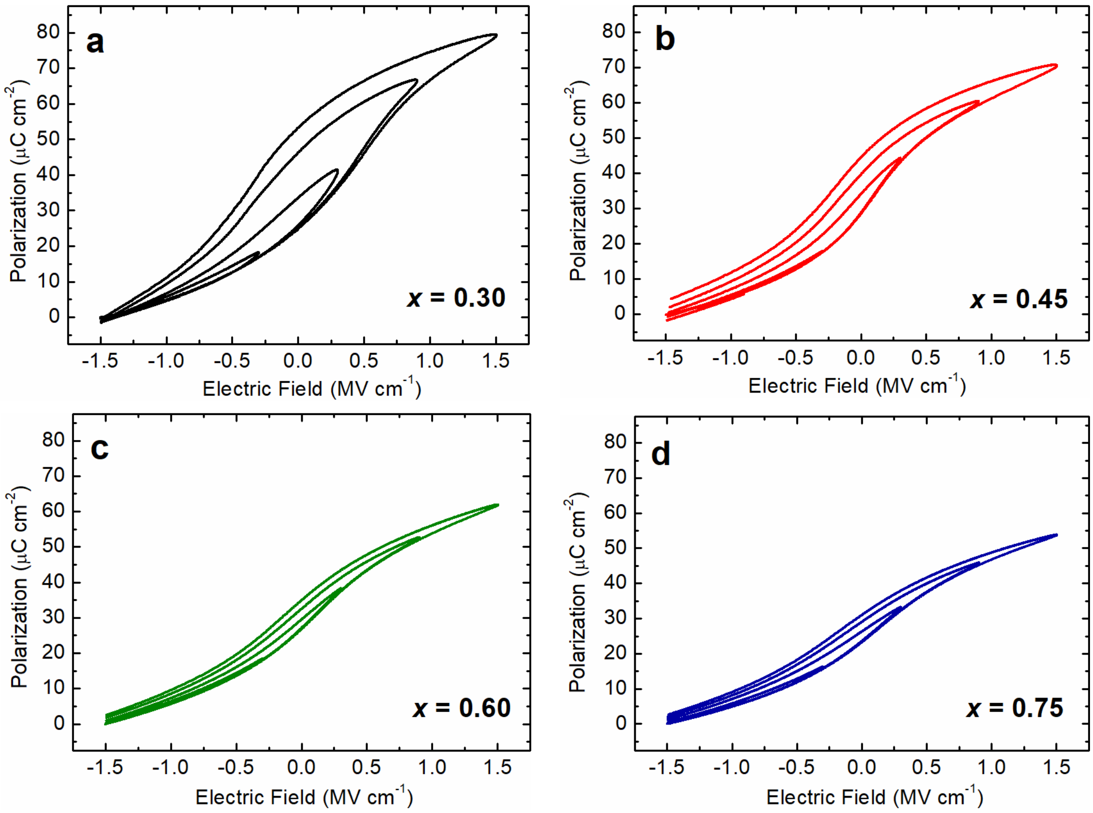

Supplementary Figure 9. FORC loops of the BFSTO films. (**a**) x = 0.30; (**b**) x = 0.45; (**c**) x = 0.60 and (**d**) x = 0.75. Only 5 out of the total 50 loops are shown for clarity.

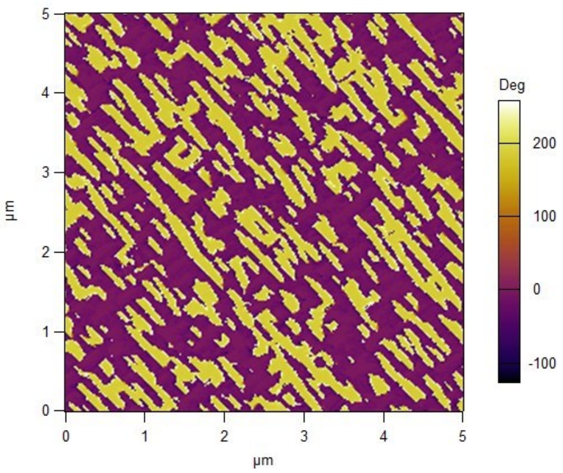

Supplementary Figure 10. Piezoelectric force microscope (PFM) image. A PFM image of the domain configuration of pure BFO film, showing the micrometer-sized ferroelectric domains.

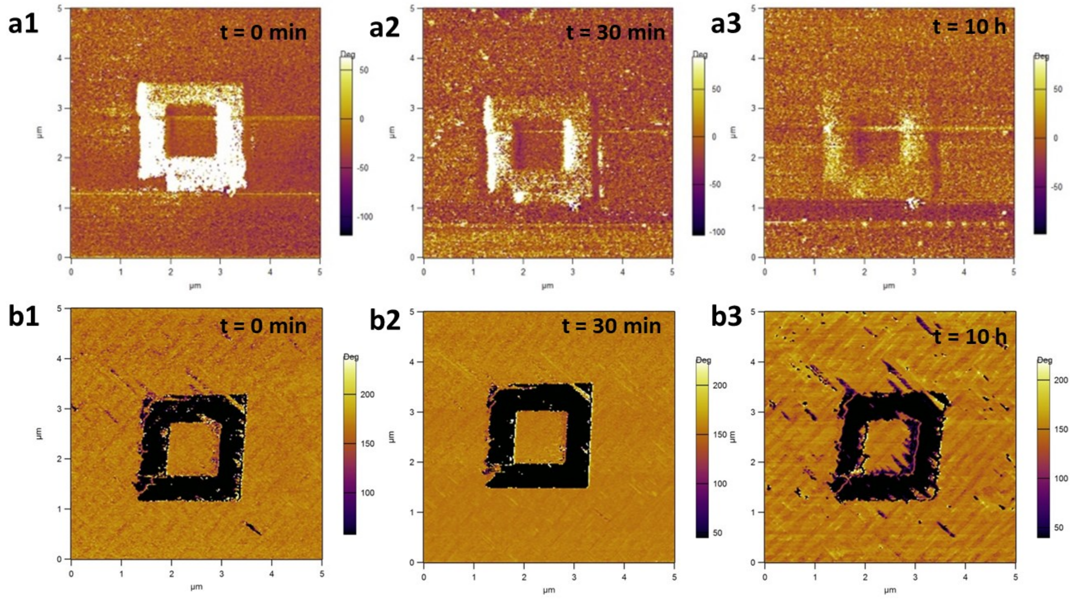

Supplementary Figure 11. Domain switching properties. PFM images of the domain configuration of (**a**) BFSTO film with x = 0.60 and (**b**) pure BFO film after various periods of time after being poled with 20 V in the annular area.

Supplementary Note 1. Decoupling of conduction and ferroelectric loss.

To decouple the different contribution sources of hysteresis loss, a reasonable assumption is given that the remnant polarization P_r at -50 ^{o}C is all originated from ferroelectric loss and the increased part at higher temperatures (i.e., $P_{cond} = P_r(T) - P_r(T = -50\ ^{o}C)$) all comes from the conduction loss; therefore the effective conductivity σ_{eff} and the conduction loss U_{cond} can be calculated by the following equations:

$$P_{\mathrm{cond}} = \frac{1}{2}\sigma_{eff}ET, \qquad (1)$$

$$U_{cond} = \frac{1}{3}\sigma_{eff}E^2T, \qquad (2)$$

where T is the period of the applied electric field. The ferroelectric loss is then obtained by subtracting U_{cond} from the total hysteresis loss. The results in Supplementary Figure 7 show that the incorporation of STO efficiently suppresses the conduction loss, especially at higher temperatures, leading to the thermal stabilization of energy performance for $x = 0.60$ and 0.75.
